# Hazardous Effect of Low-Dose Aspirin in Patients with Predialysis Advanced Chronic Kidney Disease Assessed by Machine Learning Method Feature Selection

**DOI:** 10.3390/healthcare9111484

**Published:** 2021-10-31

**Authors:** Ming-Hsien Tsai, Hung-Hsiang Liou, Yen-Chun Huang, Tian-Shyug Lee, Mingchih Chen, Yu-Wei Fang

**Affiliations:** 1 Division of Nephrology, Department of Internal Medicine, Shin-Kong Wu Ho-Su Memorial Hospital, Taipei 11101, Taiwan; chaosmyth.tw@gmail.com; 2 Department of Medicine, Fu-Jen Catholic University School of Medicine, New Taipei City 242062, Taiwan; 3 Division of Nephrology, Department of Internal Medicine, Hsin-Jen Hospital, New Taipei City 24243, Taiwan; hh258527@ms23.hinet.net; 4 Graduate Institute of Business Administration, College of Management, Fu Jen Catholic University, New Taipei City 24243, Taiwan; hivicky92@gmail.com (Y.-C.H.); 036665@mail.fju.edu.tw (T.-S.L.); 5 AI Development Center, Fu Jen Catholic University, New Taipei City 24243, Taiwan

**Keywords:** chronic kidney disease, real-world evidence, machine learning, aspirin, nonsteroidal anti-inflammatory drugs, dialysis, feature selection, machine learning

## Abstract

*Background*: Low-dose aspirin (100 mg) is widely used in preventing cardiovascular disease in chronic kidney disease (CKD) because its benefits outweighs the harm, however, its effect on clinical outcomes in patients with predialysis advanced CKD is still unclear. This study aimed to assess the effect of aspirin use on clinical outcomes in such group. *Methods*: Patients were selected from a nationwide diabetes database from January 2009 to June 2017, and divided into two groups, a case group with aspirin use (*n* = 3021) and a control group without aspirin use (*n* = 9063), by propensity score matching with a 1:3 ratio. The Cox regression model was used to estimate the hazard ratio (HR). Moreover, machine learning method feature selection was used to assess the importance of parameters in the clinical outcomes. *Results*: In a mean follow-up of 1.54 years, aspirin use was associated with higher risk for entering dialysis (HR, 1.15 [95%CI, 1.10–1.21]) and death before entering dialysis (1.46 [1.25–1.71]), which were also supported by feature selection. The renal effect of aspirin use was consistent across patient subgroups. Nonusers and aspirin users did not show a significant difference, except for gastrointestinal bleeding (1.05 [0.96–1.15]), intracranial hemorrhage events (1.23 [0.98–1.55]), or ischemic stroke (1.15 [0.98–1.55]). *Conclusions*: Patients with predialysis advanced CKD and anemia who received aspirin exhibited higher risk of entering dialysis and death before entering dialysis by 15% and 46%, respectively.

## 1. Introduction

Chronic kidney disease (CKD) is a global health burden, with a prevalence of up to 15% [1,2]. Compared with the general population, individuals with CKD are at an increased risk for cardiovascular disease (CVD)-related death, which is a leading cause of mortality in the population with CKD [3]. Therefore, preventive measures for CVD are of great importance in patients with CKD. Previous studies have demonstrated that efforts to lower blood pressure, lipid levels, uric acid levels, and sugar levels and use of renin–angiotensin–aldosterone system blocker and antiplatelet medication are effective in reducing the risk of CVD in patients with CKD [3,4,5].

Aspirin, a type of antiplatelet medication, is recommended by the 2019 American College of Cardiology/American Heart Association Guideline as a primary prevention strategy in individuals to reduce the risk of CVD [6]. One of the largest individual (*n* = 18,790) randomized control trials (RCTs) on primary prevention (the Hypertension Optimal Treatment [HOT] trial) showed a significant 15% overall reduction in major cardiovascular events [7]. Moreover, the follow-up post-hoc analysis of HOT study showed that there was a positive association between the CV benefit of aspirin and CKD levels, indicating that aspirin could provide more benefit of CVD prevention as renal function declines [8,9]. A recent meta-analysis concluded that low-dose aspirin can reduce the major cardiovascular events (MACE) [10].

Low-dose aspirin (100 mg) also belongs to the class of nonsteroidal anti-inflammatory drugs (NSAIDs), which have long been regarded as dangerous for use in patients with CKD because of their risk for nephrotoxicity by inhibiting renal prostaglandin (PG) excretion and inducing acute interstitial nephritis [11]. NSAIDs should be avoided in patients with CKD by The National Kidney Foundation’s Kidney Disease Outcomes Quality Initiative [12]. Current available data on the long-term effects of low-dose aspirin on the progression of CKD in humans are inconclusive. However, three main RCT studies had reported the aspirin did not affect the renal function in patients with CKD, including the first United Kingdom Heart and Renal Protection (UK-HARP-1) trial [13], HOT study with post hoc analysis [9], and Aspirin for Primary Prevention of CVD and Renal Disease Progression (AASER) study [14]. Therefore, aspirin is still recommended for patients with CKD for CVD prevention even though it is a NSAID [12].

In clinical practice, one question always asked by nephrologists is as follows: Can patients with advanced CKD (estimated glomerular filtration rate [eGFR] < 15 mL/min/1.73 m^2^), who are more vulnerable to kidney damage, still benefit from aspirin use? Hsiao et al. tried to answer this question and reported that aspirin use in advanced CKD (eGFR < 15 mL/min/1.73 m^2^) had a higher risk of entering dialysis and no benefit for mortality using 1 million beneficiary data randomly sampled from the registry for beneficiaries of the Taiwan National Health Insurance Research Datasets (NHIRD) in 2005 [14]. However, the sample size was small (230 aspirin users), and the baseline discrepancy between aspirin user and nonusers was not handled well.

Therefore, it is still unclear whether patients with advanced CKD can benefit from aspirin use. This study aimed to evaluate the benefit and risk of aspirin use in patients with predialysis advanced CKD (eGFR < 15 mL/min/17.3 m^2^) using a nationwide database in Taiwan.

## 2. Materials and Methods

### 2.1. Data Sources and Research Samples

The National Health Insurance (NHI) system was launched in March 1995. This program covers >99% of beneficiaries (approximately 23 million individuals) in Taiwan. NHIRD provides all medical information and regularly collects records of an individual’s admissions and outpatient visits. Included characteristics were demographics, institutions, initiation date, and total expenditure from hospitals and clinics. The NHI diagnosis code was defined by the International Classification of Diseases, 9th Revision, Clinical Modification (ICD-9-CM). The study of ICD-10-CM started in 1983 and was completed in 1992. The NHI Administration of Taiwan has been fully adopted since 1 January 2016. According to rigorous secrecy guidelines, personal information of all beneficiaries was de-identified and anonymous in the NHIRD data for research. It is to ensure privacy. This study was performed in accordance with the principles of the Declaration of Helsinki and was approved by the institutional review board (IRB) of Fu Jen Catholic University (Approval number: C104016). The requirement for informed consent was waived by the IRB of Fu Jen Catholic University because the NHIRD data were anonymized and de-identified prior to analysis in this study.

### 2.2. Study Population and Exclusion Criteria

Patients with predialysis advanced CKD were defined as those patients with CKD who received medication of erythropoietin stimulating agent (ESA) at least two times, indicating that their serum creatinine levels were >6 mg/dL (eGFR < 15 mL/min/17.3 m^2^) and hematocrit levels were <28% between 1 January 2009 and 30 June 2017 (*n* = 158,738). We adopted this definition of advanced CKD by referencing the study conducted by Hsu et al. [15]. Patients who had received dialysis (peritoneal dialysis or hemodialysis) or transplant kidney before ESA use (*n* = 49,923), were aged <18 or >100 years (*n* = 402), had missing information (*n* = 173), and died or had dialysis within three months after receiving ESA (*n* = 14,494) were excluded from the study.

**Figure 1:**
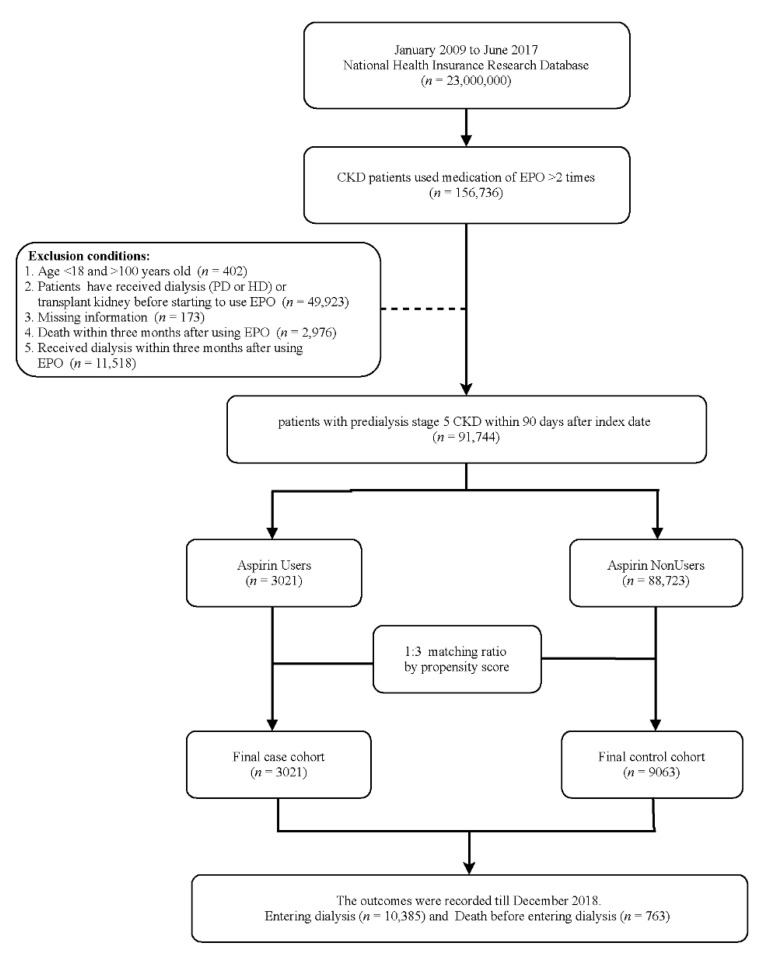
Schema of patient enrollment in the study.

A total of 91,744 patients were divided into the case and comparison groups. Patients who used aspirin within 90 days after use of ESA (*n* = 3021) were defined as the case group, and the comparison group was selected from patients who did not use aspirin within 90 days after use of ESA (*n* = 88,723). To reduce baseline difference between two groups, we used 1:3 propensity score matched with age, sex, Charlson comorbidity index scores [16], comorbidities, and medications. Ultimately, we evaluated aspirin users (*n* = 3021) and 9063 patients in the matched comparison group (Figure 1).

### 2.3. Clinical Outcomes

This study was started from the third month after use of ESA (index day), and follow-up was conducted until the occurrence of clinical events, including dialysis, death, all-cause hospitalization, gastrointestinal bleeding, ischemic stroke, intracranial hemorrhage, and MACE, of which the composites are myocardial infarction, cerebrovascular disease, heart failure, and arrhythmia, or end of the study (31 December 2017). The baseline characteristics of comorbidities and medications were considered if they might affect the relationship between low-dose aspirin use and clinical outcomes. Comorbidities were confirmed by the criteria of at least two visits to the outpatient department or one admission in one year before the index date. Medications of interest were defined as those that the patients used 30 days in three months before the index day. The codes for characteristics (Table S1) including sex, age, and commodities; medications (Table S2) including anti-hypertension agents, potassium diuretic, metformin, insulin, lipid-lowering agents, and pain killers; and clinical outcomes (Table S1) are provided as Supplementary Files.

### 2.4. Feature Selection

Huang et al., found that feature selection (FS) is a necessary preprocessing step that can help identify which variables could affect survival and medical expense and it has been applied in lot of medical informatics and research [17,18]. Through FS we can more easily understand which predicters can make predictions more and more accurate. This research used three different FS methods to estimate the importance of parameters on entering dialysis and death before entering dialysis of patients with advanced CKD and anemia. Three different machine learning models were used to evaluate which variables can affect the outcomes more, including logistic regression (LGR), random forest (RF), and eXtreme Gradient Boosting (XGboost). The important variables selected using each method were ranked.

### 2.5. Statistical Analysis

The demographic variables were expressed as number (%) for categorical variables and mean ± standard deviation for continuous variables. Chi-square tests were used to compare the proportion between groups, and t-test was used for the means of continuous variables. A propensity score matching with a 1:3 ratio was used to eliminate the baseline discrepancy between aspirin users and nonusers [19] All analyses were conducted on an intention-to-treatment basis according to the patients’ initial aspirin use without consideration of the subsequent regimen change. The Kaplan–Meier curve method was conducted for estimating the event-free curves and tested using a log-rank test. A Cox proportional regression model was adopted to estimate the hazard ratios (HR) and 95% confidence interval (CI) for the risk of clinical outcomes, including entering dialysis, death before entering dialysis, hospitalization, gastrointestinal bleeding, heart failure, intracranial hemorrhage, and MACE. The assumption of proportional hazard was not violated by testing for interaction between time and variables. Statistically significance was interpreted as two-tailed *p*-value < 0.05. All analysis results were performed by SAS version 9.4 (SAS Institute, Cary, NC, USA). The method of feature selection was adopted using R software (version 3.4.3; R Foundation for Statistical Computing, Vienna, Austria).

## 3. Results

### 3.1. Patient Characteristics

Finally, we enrolled 12,084 patients with predialysis advanced CKD and anemia in the present study (Figure 1). The case and control groups were matched by propensity score with a ratio of 1:3. Among this population, 3021 patients had at least one prescription of aspirin within 90 days after the first ESA prescription. The mean age of aspirin users was 65.8 years, of whom 56.8% were men, 55.8% had diabetes mellitus, 32.3% had coronary artery disease, and 21.3% had stroke history. Moreover, 43.5% of patients were from northern Taiwan (Table 1). Before matching, aspirin users had more comorbidities than aspirin nonusers. However, the baseline characteristics between aspirin users and nonusers had no significant difference after matching (Table 1).

### 3.2. Nephrotoxcitiy of Aspirin in Patients with Predialysis Advanced CKD

In Table 2, the total follow-up was 18,670 person-years (PY) during the study period; 10,385 patients (85.9%) underwent dialysis, and 763 patients (6.3%) died before entering dialysis. Before matching, the incidence rates of entering dialysis was 0.64/PY for aspirin users and 0.50/PY for nonusers. The incidence rates of death before entering dialysis was 0.06/PY for aspirin users and 0.04/PY for nonusers. After matching, the incidence rate of entering dialysis was 0.53/PY for aspirin nonusers, and the incidence rate of death before entering dialysis was 0.06/PY for aspirin nonusers.

**Figure 2:**
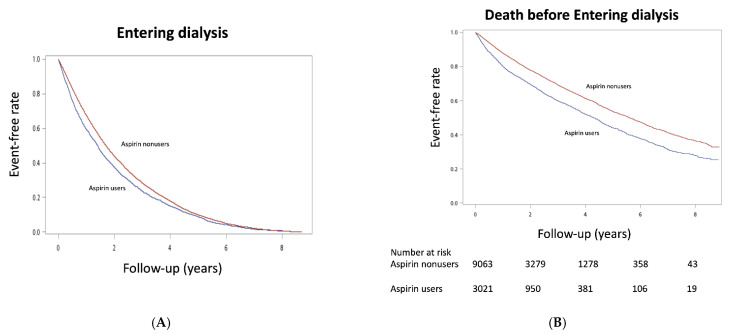
Kaplan–Meier cumulative event-free plots of (**A**) entering dialysis and (**B**) death before entering dialysis in the study population according to whether aspirin was used. A significant difference in entering dialysis (log-rank test, χ2 = 68.5, *p* < 0.001) and death before entering dialysis (log-rank test, χ2 = 104, *p* < 0.001) was noted between groups.

The Kaplan–Meier event-free curves for entering dialysis (Figure 2A) and dialysis or death before entering dialysis (Figure 2B) among aspirin users compared with nonusers were both significant (*p* < 0.001) after matching. This finding indicated that aspirin users had a higher risk of initiating long-term dialysis. As shown in Table 2, we found that treatment with aspirin in patients with stage 5 CKD significantly increased the risk for entering dialysis and death before entering dialysis before matching, with identical crude HRs of 1.23 (95%CI, 1.18–1.28) and 1.49 (95%CI, 1.30–1.70). The hazardous effects of aspirin use on long-term dialysis (HR, 1.15 [95%CI, 1.10–1.21]) and death before entering dialysis (1.46 [1.25–1.71]) persisted after propensity score matching.

### 3.3. Aspirin Effect on Other Clinical Outcomes in Patients with Predialysis Advanced CKD

Table 2 also shows the incidence rates of all-cause hospitalization (0.375/PY vs. 0.337/PY), gastrointestinal bleeding (0.089/PY vs. 0.081/PY), ischemic stroke (0.012/PY vs. 0.010/PY), intracranial hemorrhage (0.012/PY vs. 0.010/PY), and all-cause mortality (0.19/PY vs. 0.13/PY) between aspirin users and nonusers in patients with predialysis advanced CKD before matching. The crude HR is significant only in hospitalization (1.12; 95%CI, 1.07–1.16), gastrointestinal bleeding (1.10; 95%CI, 1.01–1.19) and all-cause mortality (1.51; 95%CI, 1.43–1.59). However, after matching, only all-cause hospitalization (HR, 1.06; 95%CI, 1.02–1.11) and all-cause mortality (HR, 1.37; 95%CI, 1.29–1.45) showed significance.

### 3.4. Subgroup Analysis

**Figure 3:**
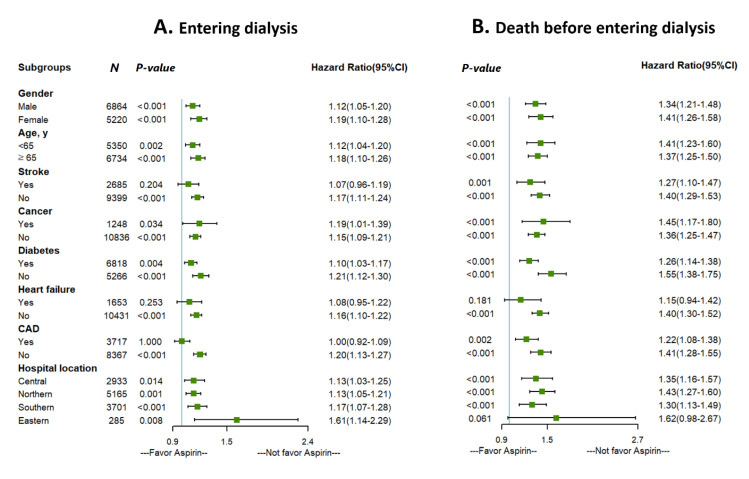
Subgroup analysis of the effect of aspirin use on (**A**) entering dialysis and (**B**) death before entering dialysis.

We conducted a series of stratified analyses to test the reliability of our analyses (Figure 3). Increased HRs of entering dialysis and death before entering dialysis among patients with predialysis advanced CKD in favor of aspirin nonusers were consistent across almost all patient subgroups. However, those with history of stroke, heart failure, and coronary artery disease showed no significant difference for entering dialysis between aspirin users and nonusers, while those with heart failure showed no significant difference for death before entering dialysis.

### 3.5. Feature Selection of Important Parameters

**Figure 4:**
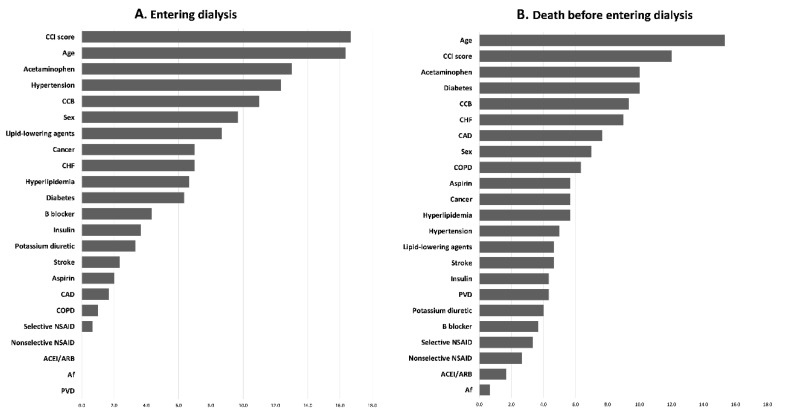
Ranking of importance of parameters for (**A**) entering dialysis and (**B**) death before entering dialysis.

The importance ranking of parameters for entering dialysis and death before entering dialysis by machine learning methods were shown in Supplementary Data (Table S3). Figure 4 shows the ranking by the average scores of LGR, RF, and XGboost, in which age and CCI score were the top two in both entering dialysis and death before entering dialysis. The parameter, aspirin, was ranked 16 of 23 for entering dialysis and 10 of 23 for death before entering dialysis.

## 4. Discussion

In this present nationwide study, we evaluated the effectiveness of low-dose aspirin in the population of predialysis advanced CKD. We found significant risk for entering dialysis and death before entering dialysis in patients with predialysis advanced CKD receiving aspirin therapy. Aspirin use in most subgroups of patients could have similar risk elevation. Furthermore, aspirin use seems to have no significant benefit in subsequent clinical outcomes of MACE and ischemic stroke. Our study not only extends the current knowledge in the field but also alerts us on the effectiveness of aspirin use in patients with advanced CKD (stage 5).

In the kidney, cyclooxygenase (COX) enzymes, including COX-1 and COX-2, are locally produced at many sites, including the macula densa, vascular endothelium, medulla, and interstitium. The COX enzymes can enhance the production of PGs, in which renal PGs are primarily vasodilators increasing the renal plasma flow and are crucial for kidney homeostasis [20]. In healthy patients, PGs play a little role in renal hemodynamics, but it became more important to preserve the GFR while the renal function declines (eGFR <60 mL/min/1.73 m^2^) [21]. NSAIDs, including aspirin, can inhibit the activity of COX enzymes and thereby induce renal ischemia and decline in GFR [22]. A low-dose aspirin (<100 mg) has a higher affinity with COX-1 than COX-2 [23].

However, current available high-quality studies of RCT on the long-term effects of low-dose aspirin on the progression of CKD all favor aspirin use in such population with CKD [9,13,24], in which this phenomenon may be due to the suppression of thromboxane B2 production caused by aspirin [25,26]. UK-HARP-1 trial showed that low-dose aspirin use for 1 year in a mixed population of patients with native kidney CKD or kidney transplantation was not associated with faster progression of CKD [13]. Its shortage is that the follow-up time is insufficient to observe the events. Subsequently, in the post-hoc subgroup analysis of the HOT trial, Jardine et al. reported that aspirin use did not affect renal function in the overall study population, but the effects of low-dose aspirin on the renal outcome in patients within eGFR categories were not observed [9]. The HOT trial mainly included patients with diastolic hypertension, and only 2.9% had an eGFR <45 mL/min/1.73 m^2^. The recent study showed that low-dose aspirin can prevent myocardial infarction and slow the rate of progression of renal function in patients with CKD (eGFR = 15–60 mL/min/1.73 m^2^) [24]. However, the sample was relatively small. Therefore, aspirin is recommended in patients with early to moderate CKD, but its benefit is undetermined for patients with advanced CKD (eGFR <15 mL/min/1.73 m^2^), wherein this population is always excluded from RCTs due to their vulnerability.

In our study using whole population data, bleeding events, including gastrointestinal bleeding and intracranial hemorrhage, showed no significant association with aspirin use after propensity score matching, indicating that aspirin use is safe from the bleeding concern in patients with advanced CKD [27], of which this finding was similar to those of studies applying aspirin to patients with early to moderate CKD [9,13,24,28,29]. We also did not observe the benefit of aspirin use in prevention ischemic stroke events, which was consistent with a meta-analysis study on aspirin use in patients with CKD [29].

For our main purpose, we found that aspirin users in patients with predialysis advanced CKD had an increased risk for entering dialysis, mortality, and hospitalization, of which the findings were consistent with the study conducted by Hsiao et al. [14]. An interesting finding is that low-dose aspirin had no benefit but increased the risk of MACE in the population with advanced CKD. The results of the KNOW-CKD study showed that low-dose aspirin was associated with an increased risk of CVD in patients with low body weight (<60 kg) [30]. The hypothesis may be that the effect of aspirin is attenuated in patients with advanced CKD, with high treatment platelet reactivity [31], leading to insufficient heart protection or stronger affinition for COX-2, resulting in thrombotic and ischemic events in the heart, in whom COX-1 is responsible for platelet aggregation and vasoconstriction and COX-2 is for vasodilation and inhibition of platelet aggregation [31,32]. Moreover, the other possible explanation is that there are some complex imbalances of the clotting system, platelet function, and fibrinolytic system in advanced renal failure [33,34], reducing the protective effect of low-dose aspirin in thrombotic events and may enchaining the harmful effect of low-dose aspirin in clinical outcomes. However, the real pathophysiology of the hazardous effect of aspirin in the patient with advanced CKD is still needed the further exploring.

In our study, aspirin provides more harm than benefit to those with advanced CKD. Aspirin use was an important factor for entering dialysis (16th of 23) and death before entering dialysis (10th of 23) using machine learning methods. However, we cannot establish a strict conclusion that aspirin is contraindicated for patients with predialysis CKD due to our retrospective cohort study design. A large-scale RCT is needed to answer this question.

There are several strengths of this study. First, we used a nationwide database to conduct the study, indicating that the inference of our study has generalization. Second, the sample size and events were adequate to obtain sufficient inference. Third, we tried to use the machine learning method to assess the importance of aspirin use in the clinical outcomes in patients with advanced CKD. Despite its strengths, there are several limitations of this study. First, some clinical information, such as biochemical data and blood pressure, is not available in the NHIRD. Second, we cannot determine whether the study patients had regular drug compliance because aspirin exposure was based on prescription information only. This bias may be alleviated using the statistical method of intention to treat under the assumption of a random misclassification. Third, this was not an RCT, so the bias of confounding by indication was the major concern due to unbalanced underline [35]. However, an RCT is difficult to conduct in these population due to their vulnerability. Therefore, we tried to use propensity score matching to eliminate this baseline imbalance in our observational cohort study.

## 5. Conclusions

In conclusion, although low-dose aspirin is recommended for the prevention of CVD in patients with CKD, our results show that the use of low-dose aspirin in patients with predialysis advanced CKD (eGFR <15 mL/min/1.73 m^2^) has potentially harmful effects, as it increases renal progression and death. Further large-scale RCTs are needed to confirm the effectiveness of low-dose aspirin therapy on these patients.

## Figures and Tables

**Table 1:** Demographic and clinical characteristics of patients with predialysis advance CKD.

	Before Matching			After Matching		
Variables	Aspirin User (*n* = 3021)	Aspirin Nonuser (*n* = 88,723)	*p* Value	Aspirin User (*n* = 3021)	Aspirin Nonuser (*n* = 9063)	*p* Value
**Gender**						
Male (%)	1716 (56.8)	45,893 (51.7)	<0.001	1716 (56.8)	5148 (56.8)	1
Female (%)	1305 (43.2)	42,830 (48.3)		1305 (43.2)	3915 (43.2)	
Age	65.8 ± 12.9	64.6 ± 13.8	<0.001	65.8 ± 12.9	65.5 ± 13.1	0.284
**Age group**			<0.001			0.976
<50 (%)	995 (32.9)	29,231 (33.0)		995 (33.0)	2988 (33.0)	
50–64 (%)	345 (11.4)	12,518 (14.1)		345 (11.4)	1022 (11.3)	
≥65 (%)	1681 (55.6)	46,974 (53.0)		1681 (55.6)	5053 (55.8)	
**Comorbidities**						
Hypertension (%)	609 (20.)	17,177 (19.4)	0.274	609 (20.2)	1979 (21.8)	0.051
Diabetes mellitus (%)	1685 (55.8)	44,498 (50.2)	<0.001	1685 (55.8)	5133 (56.6)	0.408
Hyperlipidemia (%)	1313 (43.5)	37,650 (42.4)	0.261	1313 (43.5)	4307 (47.5)	<0.001
CAD (%)	977 (32.3)	22,711 (25.6)	<0.001	977 (32.3)	2740 (30.2)	0.029
CHF (%)	1234 (40.9)	27,283 (30.8)	<0.001	1234 (40.9)	3708 (40.9)	0.948
Stroke (%)	655 (21.3)	17,615 (19.9)	0.047	655 (21.3)	2041 (22.5)	0.168
PVD (%)	297 (9.8)	8361 (9.4)	0.451	297 (9.8)	966 (10.7)	0.197
COPD (%)	576 (19.1)	17,083 (19.3)	0.796	576 (19.1)	1945 (21.5)	0.005
Cancer (%)	297 (9.8)	9468 (10.7)	0.14	297 (9.8)	951 (10.5)	0.3
Atrial fibrillation (%)	143 (4.7)	3528 (4.0)	0.036	143 (4.7)	409 (4.5)	0.614
CCIS	4.98 ± 3.1	4.69 ± 3.2	<0.001	4.98 ± 3.1	4.91 ± 3.1	0.23
**Hospital area**						
Central (%)	740 (24.5)	21,066 (23.7)	0.021	740 (24.5)	2193 (24.2)	0.175
Northern (%)	1329 (44.0)	37,313 (42.1)		1329 (44.0)	3836 (42.3)	
Southern (%)	890 (29.5)	28,253 (31.8)		890 (29.5)	2811 (31.0)	
Eastern (%)	62 (2.1)	2091 (2.4)		62 (2.1)	223 (2.5)	
**Medications**						
ACEI/ARB (%)	304 (10.1)	5732 (6.5)	<0.001	304 (10.1)	901 (9.9)	0.847
β blockers (%)	1701 (56.3)	41,354 (46.6)	<0.001	1701 (56.3)	5100 (56.3)	0.974
CCB (%)	2171 (71.9)	62,618 (70.6)	0.126	2171 (71.9)	6663 (73.5)	0.075
Potassium diuretic (%)	112 (3.7)	2596 (2.9)	0.012	112 (3.7)	282 (3.1)	0.11
Metformin (%)	27 (0.9)	478 (0.5)	0.009	27 (0.9)	52 (0.6)	0.058
Insulin (%)	897 (29.7)	20,640 (23.3)	<0.001	897 (29.7)	2689 (29.7)	0.981
Lipid-lowering agents (%)	927 (30.7)	22,080 (24.9)	<0.001	927 (30.7)	2782 (30.7)	0.99
Nonselective NSAID (%)	683 (22.6)	17,208 (19.4)	<0.001	683 (22.6)	2052 (22.6)	0.97
Selective NSAID (%)	231 (7.7)	5736 (6.5)	0.009	231 (7.7)	638 (7.0)	0.263
Acetaminophen (%)	1568 (51.9)	40,452 (45.6)	<0.001	1568 (51.9)	4706 (51.9)	0.983

Abbreviation: CKD: chronic kidney disease; CAD: coronary artery disease; CHF: congestive heart failure, PVD: peripheral vascular disease; COPD: chronic obstructive pulmonary disease: CCIS: Charlson comorbidity index score; ACEI: angiotensin converting enzyme inhibitors; ARB: angiotensin receptor blocker; CCB: calcium channel blocker; NSAID: non-steroidal anti-inflammatory drug.

**Table 2:** Risk of clinical outcomes in patients with predialysis advance CKD comparing aspirin Users vs. Nonusers.

Clinical Outcomes	Before Matching						After Matching					
	Aspirin Users (*n* = 3021)		Aspirin Nonusers (*n* = 88,723)		Aspirin Users vs. Nonusrs		Aspirin Users (*n* = 3021)		Aspirin Nonuser (*n* = 9063)		Aspirin Users vs. Nonusrs	
	Events	IR	Events	IR	Crude HR (95%CI)	*p* Value	Events	IR	Events	IR	HR (95%CI)	*p* Value
Dialysis	2616	64.0	74,777	49.8	1.23 (1.18–1.28)	<0.001	2616	64.0	7769	53.2	1.15 (1.10–1.21)	<0.001
Dead before dialysis	228	5.6	5344	3.6	1.49 (1.30–1.70)	<0.001	228	5.6	535	3.7	1.46 (1.25–1.71)	<0.001
All-cause Hospitalization	2427	37.5	70,969	33.7	1.12 (1.07–1.16)	<0.001	2427	37.5	7373	35.1	1.06 (1.02–1.11)	0.009
GI bleeding	640	8.9	19,683	8.1	1.10 (1.01–1.19)	0.021	640	8.9	2027	8.5	1.05 (0.96–1.15)	0.280
Ischemic stroke	100	1.2	2905	1.0	1.16 (0.95–1.42)	0.141	100	1.2	290	1.0	1.15 (0.92–1.45)	0.217
ICH	102	1.2	2883	1.0	1.20 (0.98–1.46)	0.073	102	1.2	278	1.0	1.23 (0.98–1.55)	0.071
All-cause mortality	1460	19.0	32,706	13.2	1.51 (1.43–1.59)	<0.001	1460	19.0	3557	12.4	1.37 (1.29–1.45)	<0.001
MACE	2351	27.9	58,956	20.7	1.29 (1.24–1.35)	<0.001	2351	27.9	6613	26.6	1.15 (1.10–1.20)	<0.001

Abbreviation: IR: Incidence rate; PY: Person-year; Rate incidence per 100 PYs, HRs: Hazard ratio; CKD: chronic kidney disease; CI: confidence interval; GI: gastrointestinal; ICH, Intracranial hemorrhage; MACE: major adverse cardiac event.

## Data Availability

Due to the General Data Protection Regulation, NHIRD presented in this study are not available on request from the corresponding author.

## Supplementary Materials

The following are available online at https://www.mdpi.com/article/10.3390/healthcare9111484/s1, Table S1: Code for comorbidities, Table S2: Code for drugs, Table S3: the score of ML.

## References

[1] Tsai M.H., Hsu C.Y., Lin M.Y., Yen M.F., Chen H.H., Chiu Y.H., Hwang S.J. (2018). Incidence, Prevalence, and Duration of Chronic Kidney Disease in Taiwan: Results from a Community-Based Screening Program of 106,094 Individuals. Nephron.

[2] Lv J.C., Zhang L.X. (2019). Prevalence and Disease Burden of Chronic Kidney Disease. Adv. Exp. Med. Biol..

[3] Provenzano M., Coppolino G., Faga T., Garofalo C., Serra R., Andreucci M. (2019). Epidemiology of cardiovascular risk in chronic kidney disease patients: The real silent killer. Rev. Cardiovasc. Med..

[4] Major R.W., Cheng M.R.I., Grant R.A., Shantikumar S., Xu G., Oozeerally I., Brunskill N.J., Gray L.J. (2018). Cardiovascular disease risk factors in chronic kidney disease: A systematic review and meta-analysis. PLoS ONE.

[5] Gansevoort R.T., Correa-Rotter R., Hemmelgarn B.R., Jafar T.H., Heerspink H.J., Mann J.F., Matsushita K., Wen C.P. (2013). Chronic kidney disease and cardiovascular risk: Epidemiology, mechanisms, and prevention. Lancet.

[6] Arnett D.K., Blumenthal R.S., Albert M.A., Buroker A.B., Goldberger Z.D., Hahn E.J., Himmelfarb C.D., Khera A., Lloyd-Jones D., McEvoy J.W. (2019). 2019 ACC/AHA Guideline on the Primary Prevention of Cardiovascular Disease: Executive Summary: A Report of the American College of Cardiology/American Heart Association Task Force on Clinical Practice Guidelines. J. Am. Coll. Cardiol..

[7] Hansson L., Zanchetti A., Carruthers S.G., Dahlof B., Elmfeldt D., Julius S., Menard J., Rahn K.H., Wedel H., Westerling S. (1998). Effects of intensive blood-pressure lowering and low-dose aspirin in patients with hypertension: Principal results of the Hypertension Optimal Treatment (HOT) randomised trial. HOT Study Group. Lancet.

[8] Ruilope L.M., Salvetti A., Jamerson K., Hansson L., Warnold I., Wedel H., Zanchetti A. (2001). Renal function and intensive lowering of blood pressure in hypertensive participants of the hypertension optimal treatment (HOT) study. J. Am. Soc. Nephrol..

[9] Jardine M.J., Ninomiya T., Perkovic V., Cass A., Turnbull F., Gallagher M.P., Zoungas S., Lambers Heerspink H.J., Chalmers J., Zanchetti A. (2010). Aspirin is beneficial in hypertensive patients with chronic kidney disease: A post-hoc subgroup analysis of a randomized controlled trial. J. Am. Coll. Cardiol..

[10] Su X., Yan B., Wang L., Lv J., Cheng H., Chen Y. (2019). Effect of antiplatelet therapy on cardiovascular and kidney outcomes in patients with chronic kidney disease: A systematic review and meta-analysis. BMC Nephrol..

[11] Baker M., Perazella M.A. (2020). NSAIDs in CKD: Are They Safe?. Am. J. Kidney Dis..

[12] Inker L.A., Astor B.C., Fox C.H., Isakova T., Lash J.P., Peralta C.A., Kurella Tamura M., Feldman H.I. (2014). KDOQI US commentary on the 2012 KDIGO clinical practice guideline for the evaluation and management of CKD. Am. J. Kidney Dis..

[13] Baigent C., Landray M., Leaper C., Altmann P., Armitage J., Baxter A., Cairns H.S., Collins R., Foley R.N., Frighi V. (2005). First United Kingdom Heart and Renal Protection (UK-HARP-I) study: Biochemical efficacy and safety of simvastatin and safety of low-dose aspirin in chronic kidney disease. Am. J. Kidney Dis..

[14] Hsiao K.C., Huang J.Y., Lee C.T., Hung T.W., Liaw Y.P., Chang H.R. (2017). Different impact of aspirin on renal progression in patients with predialysis advanced chronic kidney disease with or without previous stroke. Eur. J. Intern. Med..

[15] Hsu T.W., Liu J.S., Hung S.C., Kuo K.L., Chang Y.K., Chen Y.C., Hsu C.C., Tarng D.C. (2014). Renoprotective effect of renin-angiotensin-aldosterone system blockade in patients with predialysis advanced chronic kidney disease, hypertension, and anemia. JAMA Intern. Med..

[16] Yang H., Chen Y.H., Hsieh T.F., Chuang S.Y., Wu M.J. (2016). Prediction of Mortality in Incident Hemodialysis Patients: A Validation and Comparison of CHADS2, CHA2DS2, and CCI Scores. PLoS ONE.

[17] Huang Y.C., Li S.J., Chen M., Lee T.S., Chien Y.N. (2021). Machine-Learning Techniques for Feature Selection and Prediction of Mortality in Elderly CABG Patients. Healthcare.

[18] Huang Y.C., Li S.J., Chen M., Lee T.S. (2021). The Prediction Model of Medical Expenditure Appling Machine Learning Algorithm in CABG Patients. Healthcare.

[19] Austin P.C. (2011). An Introduction to Propensity Score Methods for Reducing the Effects of Confounding in Observational Studies. Multivar. Behav. Res..

[20] Kim G.H. (2008). Renal effects of prostaglandins and cyclooxygenase-2 inhibitors. Electrolyte Blood Press..

[21] Patrono C., Dunn M.J. (1987). The clinical significance of inhibition of renal prostaglandin synthesis. Kidney Int..

[22] Lucas G.N.C., Leitao A.C.C., Alencar R.L., Xavier R.M.F., Daher E.F., Silva Junior G.B.D. (2019). Pathophysiological aspects of nephropathy caused by non-steroidal anti-inflammatory drugs. J. Bras. Nephrol..

[23] Ornelas A., Zacharias-Millward N., Menter D.G., Davis J.S., Lichtenberger L., Hawke D., Hawk E., Vilar E., Bhattacharya P., Millward S. (2017). Beyond COX-1: The effects of aspirin on platelet biology and potential mechanisms of chemoprevention. Cancer Metastasis Rev..

[24] Goicoechea M., de Vinuesa S.G., Quiroga B., Verde E., Bernis C., Morales E., Fernandez-Juarez G., de Sequera P., Verdalles U., Delgado R. (2018). Aspirin for Primary Prevention of Cardiovascular Disease and Renal Disease Progression in Chronic Kidney Disease Patients: A Multicenter Randomized Clinical Trial (AASER Study). Cardiovasc. Drugs Ther..

[25] Okumura M., Imanishi M., Yamashita T., Yamamura Y., Kim S., Iwao H., Tanaka S., Fujii S. (2000). Renal production of thromboxane and prostaglandins in a rat model of type 2 diabetes. Life Sci..

[26] Boffa J.J., Just A., Coffman T.M., Arendshorst W.J. (2004). Thromboxane receptor mediates renal vasoconstriction and contributes to acute renal failure in endotoxemic mice. J. Am. Soc. Nephrol..

[27] Molnar A.O., Bota S.E., Garg A.X., Harel Z., Lam N., McArthur E., Nesrallah G., Perl J., Sood M.M. (2016). The Risk of Major Hemorrhage with CKD. J. Am. Soc. Nephrol..

[28] Palmer S.C., Di Micco L., Razavian M., Craig J.C., Perkovic V., Pellegrini F., Jardine M.J., Webster A.C., Zoungas S., Strippoli G.F. (2013). Antiplatelet agents for chronic kidney disease. Cochrane Database Syst. Rev..

[29] Kim A.J., Lim H.J., Ro H., Ko K.P., Han S.Y., Chang J.H., Lee H.H., Chung W., Jung J.Y. (2014). Low-dose aspirin for prevention of cardiovascular disease in patients with chronic kidney disease. PLoS ONE.

[30] Oh Y.J., Kim A.J., Ro H., Chang J.H., Lee H.H., Chung W., Hyun Y.Y., Lee J., Kim Y.H., Han S.H. (2021). Low-dose aspirin was associated with an increased risk of cardiovascular events in patients with chronic kidney disease patients and low bodyweight: Results from KNOW-CKD study. Sci. Rep..

[31] Polzin A., Dannenberg L., Sansone R., Levkau B., Kelm M., Hohlfeld T., Zeus T. (2016). Antiplatelet effects of aspirin in chronic kidney disease patients. J. Thromb. Haemost..

[32] Solomon S.D., McMurray J.J., Pfeffer M.A., Wittes J., Fowler R., Finn P., Anderson W.F., Zauber A., Hawk E., Bertagnolli M. (2005). Cardiovascular risk associated with celecoxib in a clinical trial for colorectal adenoma prevention. N. Engl. J. Med..

[33] Penzes K., Hurjak B., Katona E., Becs G., Balla J., Muszbek L. (2020). Terminal Phase Components of the Clotting Cascade in Patients with End-Stage Renal Disease Undergoing Hemodiafiltration or Hemodialysis Treatment. Int. J. Mol. Sci..

[34] Huang M.J., Wei R.B., Wang Y., Su T.Y., Di P., Li Q.P., Yang X., Li P., Chen X.M. (2017). Blood coagulation system in patients with chronic kidney disease: A prospective observational study. BMJ Open.

[35] Kyriacou D.N., Lewis R.J. (2016). Confounding by Indication in Clinical Research. JAMA.

