# Supplementary material for: Hazardous Effect of Low-Dose Aspirin in Patients with Predialysis Advanced Chronic Kidney Disease Assessed by Machine Learning Method Feature Selection
Source: Healthcare (Basel). 2021 Oct 31;9(11):1484. doi: 10.3390/healthcare9111484 (PMC8625790; doi:10.3390/healthcare9111484)
Supplement: Supplementary file 1 [file healthcare-09-01484-s001.zip › healthcare-1397492 Supplementary/S1_Code for comorbidities.pdf]

Table S1. Code for disease

	ICD-9-CM	ICD-10-CM
Comorbidities		
Hypertension	401 – 405	I10-14
Diabetes mellitus	250	E10-E14
Hyperlipidemia	272.0–272.4	E78
CAD	410 – 414	I20-I25
CHF	428.0, 428.1, 428.9	I50
Stroke	430–438	I60- I63, I65-I69, G45-G46
PVD	250.7, 440.2-3,440.8-9, 443,444.22,444.8,447.8-9	E08.51-52, E08.59, E09.51-52, E09.59, E10.51-52, E10.59, E11.51-52, E11.59, E13.51-52, E13.59, I70.2-I70.9, I73, I74.2-I74.9, I75.011-I75.029, I75.89, I77.3, I77.89, I77.9, I79.1, I79.8
COPD	490–492, 496	J40-J44, J47
Cancer	140-208	C00.0-C96.9
Atrial fibrillation	427.31	I48
CCI score		
Myocardial infarct	410	I21-22
Congestive heart failure	428.0, 428.1, 428.9	I50

Cerebrovascular disease	430–438	I60- I63, I65-I69, G45-G46
Connective tissue disease	710,714,725	M05, M06, M32, M33.20, M33.29, M34, M35.3
Peripheral vascular disease	250.7, 440.2-3,440.8-9, 443,444.22,444.8,447.8-9	E08.51-52, E08.59, E09.51-52, E09.59, E10.51-52, E10.59, E11.51-52, E11.59, E13.51-52, E13.59, I70.2-I70.9, I73, I74.2-I74.9, I75.011-I75.029, I75.89, I77.3, I77.89, I77.9, I79.1, I79.8
Dementia	290	F03.90, F05, F01.50, F01.51
COPD	490–492, 496	J40-J44, J47
Ulcer disease	531.XX, 532.XX, 533.XX ,534.XX	K25.X, K26.X, K27.X, K28.X, K31.82, K56.60
Mild liver disease	571.2,571.4X,571.5,571.6	K70.2, K70.3X, K73.X, K74.X, K74.60, K74.69, K75.4
Hemiplegia	342	G81
Moderate-to-severe renal disease	582.0,582.1,582.2,582.4,582.81,582.89,582.9,583.0, 583.1,583.2,583.4,583.6,583.7,583.9,588.1,588.8, 588.9, 583.81,583.89,585,586,588	E10.21, E11.21, N03.0, N03.1, N03.2, N03.3, N03.4, N03.5 N03.6, N03.7, N03.8, N03.9, N05.0, N05.1, N05.2, N05.3, N05.4, N05.5, N05.6, N05.7, N05.8, N05.9, N06.0, N06.1, N06.2, N06.3, N06.4, N06.5, N06.6, N06.7, N06.8, N06.9, N07.0, N07.1, N07.2, N07.3, N07.4, N07.5, N07.6, N07.7, N07.8, N07.9, N08, N14.0, N14.1, N14.2, N14.3, N14.4, N15.0, N15.8, N15.9, N16, N17.1, N17.2, N18.4, N18.5, N18.6, N18.9, N19, N25.0, N25.1, N25.81, N25.89, N25.9
Diabetes-with-end-organ-	250.4,250.5,250.6	E08.311, E08.319, E08.321, E08.329, E08.331, E08.339,

damage		E08.341, E08.349, E08.351, E08.359, E08.36, E08.39, E09.311, E09.319, E09.321, E09.329, E09.331, E09.339, E09.341, E09.349, E09.351, E09.359, E09.36, E09.39, E10.21, E10.22, E10.29, E10.311, E10.319, E10.36, E10.39, E10.40, E10.41, E10.44, E10.49, E10.610, E10.65, E11.21, E11.22, E11.29, E11.311, E11.319, E11.321, E11.329, E11.331, E11.339, E11.341, E11.349, E11.351, E11.359, E11.36, E11.39, E11.40, E11.41, E11.42, E11.43, E11.44, E11.49, E11.65, E13.21, E13.22, E13.29, E13.311, E13.319, E13.321, E13.329, E13.331, E13.339, E13.341, E13.349, E13.351, E13.359, E13.36, E13.39, E13.42, E13.43, E13.44, E13.49
Malignancy	140-149,150-159,160-165,170,174,175,176,179,180-189,190-195,200-201,203-208	C00-C96,D45, D47.Z9, E31.22, Z51.12
Moderate or severe liver disease	456.0,456.1,456.20,572.X,456.21,I85.XX,	K72.10,K72.11,K72.90,K72.91,K766,K767,K768.1
Metastatic solid tumors	196.X,197.X,198.X, 198.8X,199.X	C45.9, C77.X, C78, C79, C7A.00, C7A.094, C7A.095, C7A.096, C7A.1, C7A.8, C7B, C80.0, C80.1, D3A.8, J91.0, R18.0, Z51.12
AIDS	0.42,0.43,0.44	B20
Clinical outcomes		
GI bleeding (with	530.7,530.82, 531-535,537.83,578	K25-K29, K31.811, K31.82, K92

Endoscope and use PPI)	Endoscopy Examination :28016C,47027CA, 47027CB,47043B, 47043C,47074B, 47074C, 47083C,49014CA<br>ATC classification system codes : A02BC	
Ischemic stroke	433-435	I63, I65-I66, G45-G46
ICH	430-432	I60-I62
MACE	410,426-427,428,430-435,437	I121-I122, I44-I49, I50, I60-I63, I65-I66
Dialysis	58001C,59019C,58020C,58021C,58022C,58023C,58024C,58025C,580029C,58002C,58009A,58009B,<br>58010A,58010B,58011A,58011AB,58011B,58011C,58012A,58012B,58017B,58017C,58026C, 58028C.	

Abbreviation:

ICD: international classification of disease; CAD: Coronary artery disease; CHF: Congestive heart failure; PVD: Peripheral vascular disease; COPD: Chronic obstructive pulmonary disease; CCI: Charlson Comorbidity Index; AIDS, Acquired immune deficiency syndrome; GI bleeding: Gastrointestinal Bleeding; ICH: Intracerebral hemorrhage; MACE: major adverse cardiovascular events
