# Supplementary material for: Hazardous Effect of Low-Dose Aspirin in Patients with Predialysis Advanced Chronic Kidney Disease Assessed by Machine Learning Method Feature Selection
Source: Healthcare (Basel). 2021 Oct 31;9(11):1484. doi: 10.3390/healthcare9111484 (PMC8625790; doi:10.3390/healthcare9111484)
Supplement: Supplementary file 1 [file healthcare-09-01484-s001.zip › healthcare-1397492 Supplementary/S2_Code for drugs.pdf]

Table S2. Code for drugs

Drug type	ATC classification system codes	Drug name
ACEI/ARB	C09A, C09B, C09C, C09D	Captopril, Enalapril, Lisinopril, Perindopril, Ramipril, Quinapril, Benazepril, Cilazapril, Fosinopril, Imidapril, Losartan, Eprosartan, Valsartan, Irbesartan, Candesartan, Telmisartan, Olmesartan, Azilsartan
Beta blocking agents	C07A, C07B, C07CA03, C07DA06	Alprenolol, Oxprenolol, Pindolol, Propranolol, Timolol, Sotalol, Nadolol, Carteolol, Bupranolol, Metoprolol, Atenolol, Acebutolol, Betaxolol, Bevantolol, Bisoprolol, Esmolol, Nebivolol, Labetalol, Carvedilol
CCB	C08C, C08D, C08E, C09BB, C09DB, C09DX,C10BX	Amlopidine, Felodipine, Isradipine, Nicardipine, Nifedipine, Nimodipine, Nisoldipine, Nitrendipine, Lacidipine, Barnidipine, Lercanidipine, Cilnidipine, Benidipine, Verapamil, Diltiazem
Potassium diuretic	C03DA, C03DB, C03EA	Potassium canrenoate, Spironolactone, Eplerenone, Amiloride, Triamterene
Biguanides	A10BA, A10BD	Metformin, Buformin
Insulin	A10A	Insulin
Statin	C10AA	Simvastatin, Lovastatin, Pravastatin, Fluvastatin, Atorvastatin, Rosuvastatin, Pitavastatin

Nonselective NSAID	M01AB, M01AE, M01AG, M01AX	Indomethacin, sulindac, tolmetin, diclofenac, alclofenac, etodolac, acemetacin, ketorolac, aceclofenac, ibuprofen, naproxen, ketoprofen, fenoprofen, fenbufen, flurbiprofen, tiaprofenic acid, alminoprofen, mefenamic acid, tolfenamic acid, flufenamic acid, meclofenamic acid, niflumic acid, benzydamine, tiaramide Hcl
Selective NSAID	M01AC, M01AH, M01AX01, M01AX17	piroxicam, piroxicam, tenoxicam, meloxicam, celecoxib, rofecoxib, etoricoxib, nabumetone, nimesulide
Acetaminophen	N02BE	paracetamol

Abbreviation:

ATC: anatomical therapeutic chemical; ACEI/ARB: Angiotensin-converting enzyme inhibitors/ Angiotensin receptor blockers; CCB: Calcium channel blockers; NSAID: Non-Steroidal Anti-Inflammatory Drug
