# Supplementary material for: Hazardous Effect of Low-Dose Aspirin in Patients with Predialysis Advanced Chronic Kidney Disease Assessed by Machine Learning Method Feature Selection
Source: Healthcare (Basel). 2021 Oct 31;9(11):1484. doi: 10.3390/healthcare9111484 (PMC8625790; doi:10.3390/healthcare9111484)
Supplement: Supplementary file 1 [file healthcare-09-01484-s001.zip › healthcare-1397492 Supplementary/S3_the score of ML.pdf]

Table S3. Ranking of feature selection on entering dialysis and death before entering dialysis in the patients with advanced CKD

Feature selection	**Entering dialysis**				**Death before Entering dialysis**			
	LGR (16)*	RF (15)	Xgboost (15)	Average score	LGR (15)	RF (22)	Xgboost (10)	Average score
Age	15	20	14	16.3	14	22	10	15.3
Sex	9	15	5	9.7	0	21	0	7.0
CCI score	16	19	15	16.7	15	12	9	12.0
Hypertension	14	11	12	12.3	0	15	0	5.0
CAD	0	5	0	1.7	6	17	0	7.7
Diabetes	1	8	10	6.3	8	18	4	10.0
Hyperlipidemia	5	7	8	6.7	1	16	0	5.7
CHF	11	4	6	7.0	4	20	3	9.0
Stroke	4	2	1	2.3	0	14	0	4.7
PVD	0	0	0	0.0	0	13	0	4.3
COPD	0	1	2	1.0	0	19	0	6.3
Cancer	10	0	11	7.0	10	1	6	5.7
Af	0	0	0	0.0	0	2	0	0.7
ACEI/ARB	0	0	0	0.0	0	5	0	1.7
β-blocker	0	9	4	4.3	0	11	0	3.7
CCB	12	12	9	11.0	11	10	7	9.3
Potassium diuretic	7	0	3	3.3	7	4	1	4.0

Insulin	6	5	0	3.7	5	8	0	4.3
Lipid-lowering agents	8	11	7	8.7	9	3	2	4.7
Nonselective NSAID	0	0	0	0.0	2	6	0	2.7
Selective NSAID	2	0	0	0.7	3	7	0	3.3
Acetaminophen	13	13	13	13.0	13	9	8	10.0
Aspirin	3	3	0	2.0	12	0	5	5.7

Abbreviation: CKD: chronic kidney disease; CCI: Charlson comorbidity index; CAD: coronary artery disease; CHF: congestive heart failure, PVD: peripheral vascular disease; COPD: chronic obstructive pulmonary disease; Af: atrial fibrillation; ACEI: angiotensin converting enzyme inhibitors; ARB: angiotensin receptor blocker; CCB: calcium channel blocker; NSAID: non-steroidal anti-inflammatory drug; LGR: logistic regression; RF: random forest; XGboost: eXtreme Gradient Boosting.

*16 important factors were determined by LGR method, which the higher score indicates that factor has more importance on determining the clinical outcome. We performed the feature selection in the method of RF and XGboost by the same manner.
